# Checklist of the family Simuliidae (Diptera) of Finland

**DOI:** 10.3897/zookeys.441.7600

**Published:** 2014-09-19

**Authors:** Jari Ilmonen

**Affiliations:** 1 Metsähallitus Natural Heritage Services, Vantaa, Finland

**Keywords:** Checklist, Finland, Diptera, Simuliidae

## Abstract

A checklist of the family Simuliidae (Diptera) is provided for Finland and recognizes 56 species. One new record has been added (*Simulium latipes*) and one name sunken in synonymy (*Simulium carpathicum*). Furthermore, *Simulium tsheburovae* is treated as a doubtful record.

## Introduction

Simuliidae is a relatively small family of nematoceran flies, comprised of 2,163 species (2,151 living and 12 fossil) world-wide (Adler and Crosskey 2014). Numerous taxonomical confusions and different views on nomenclature between eastern and western scientists have created confusion in blackfly studies for many decades. Some taxonomical issues concerning species occurring in North Europe have been resolved by recent synonymisations (Raastad et al. 2010, Adler and Crosskey 2014). Systematics and nomenclature of the enumeration follow that of Adler and Crosskey (2014), and only the most relevant synonyms used in previous checklists for Finland are listed.

The faunistic level of knowledge in Finland is good, only few changes were made since compilation of the previous checklist (Ilmonen and Adler 2008). Few additional species can be expected to be found, and many taxonomical issues have been resolved within the last two decades.

**Number of species:**

World: 2151 species (Adler and Crosskey 2014)

Europe: 230 species (Adler and Crosskey 2014)

Finland: 56 species

Faunistic knowledge level in Finland: good

## Checklist

Nematocera Dumeril, 1805

infraorder Culicomorpha Hennig, 1948

superfamily Chironomoidea Newman, 1834

**SIMULIIDAE** Newman, 1834

SIMULIINAE Newman, 1834

tribe Prosimuliini Enderlein, 1921

***HELODON*** Enderlein, 1921

*Helodon ferrugineus* (Wahlberg, 1844)

***PROSIMULIUM*** Roubaud, 1906

*Prosimulium hirtipes* (Fries, 1824)

*Prosimulium luganicum* Rubtsov, 1956

*Prosimulium macropyga* (Lundström, 1911)

*Prosimulium ursinum* (Edwards, 1935)

tribe Simuliini Newman, 1834

***CNEPHIA*** Enderlein, 1921

*Cnephia eremites* Shewell, 1952

*Cnephia pallipes* (Fries, 1824)

= *lapponica* (Enderlein, 1921)

***GRENIERA*** Doby & David, 1959

*Greniera ivanovae* Ivashchenko, 1970

***METACNEPHIA*** Crosskey, 1969

*Metacnephia bilineata* (Rubtsov, 1940)

= *saileri* (Stone, 1952)

*Metacnephia lyra* (Lundström, 1911)

= *tabescentifrons* (Enderlein, 1929)

= *trigoniformis* Yankovsky, 2003

*Metacnephia tredecimata* (Edwards, 1920)

***SIMULIUM*** Latreille, 1802

**sg. *Boophthora*** Enderlein, 1921

*Simulium erythrocephalum* (De Geer, 1776)

**sg. *Eusimulium*** Roubaud, 1906

*Simulium angustipes* Edwards, 1915

= *securiforme* (Rubtsov, 1956)

*Simulium aureum* Fries, 1824

*Simulium velutinum* (Santos Abreu, 1922)

**sg. *Boreosimulium*** Rubzov & Yankovsky, 1982

*Simulium annulus* (Lundström, 1911)

*Simulium baffinense* Twinn, 1936

*Simulium crassum* (Rubtsov, 1956)

**sg. *Hellichiella*** Rivosecchi & Cardinali, 1975

*Simulium dogieli* (Rubtsov, 1956)

*Simulium latipes* (Meigen, 1804)

*Simulium usovae* (Golini, 1987)

**sg. *Nevermannia*** Enderlein, 1921

= ***Cnetha*** Enderlein, 1921

*Simulium angustitarse* (Lundström, 1911)

*Simulium beltukovae* (Rubtsov, 1956)

= *carpathicum* (Knoz, 1961)

*Simulium bicorne* Dorogostaisky, Rubtsov & Vlasenko, 1935

*Simulium cryophilum* (Rubtsov, 1959)

= carthusiense f. brevicaulis Dorier & Grenier, 1961

*Simulium curvans* (Rubtsov & Carlsson, 1965)

*Simulium dendrofilum* (Patrusheva, 1962)

*Simulium fontinale* Radzivilovskaya, 1948

*Simulium juxtacrenobium* Bass & Brockhouse, 1990

*Simulium lundstromi* (Enderlein, 1921)

*Simulium silvestre* (Rubtsov, 1956)

*Simulium vernum* Macquart, 1826

= *pritzkowi* (Enderlein, 1926)

**sg. *Schoenbaueria*** Enderlein, 1921

*Simulium pusillum* Fries, 1824

*Simulium subpusillum* Rubtsov, 1940

**sg. *Simulium*** Latreille, 1802

*Simulium annulitarse* Zetterstedt, 1838

*Simulium argyreatum* Meigen, 1838

*Simulium frigidum* Rubtsov, 1940

*Simulium intermedium* Roubaud, 1906

*Simulium longipalpe* Beltyukova, 1955

= *curvistylus* Rubtsov, 1957

*Simulium monticola* Friederichs, 1920

*Simulium morsitans* Edwards, 1915

*Simulium murmanum* Enderlein, 1935

= *forsi* (Carlsson, 1962)

*Simulium noelleri* Friederichs, 1920

*Simulium ornatum* Meigen, 1818

*Simulium paramorsitans* Rubtsov, 1956

*Simulium posticatum* Meigen, 1838

= *austeni* Edwards, 1915

= *verecundum* misid.

*Simulium reptans* (Linnaeus, 1758)

*Simulium rostratum* (Lundström, 1911)

= *sublacustre* Davies, 1966

*Simulium rubtzovi* Smart, 1945

*Simulium transiens* Rubtsov, 1940

*Simulium truncatum* (Lundström, 1911)

*Simulium tuberosum* (Lundström, 1911)

*Simulium tumulosum* Rubtsov, 1956

*Simulium vulgare* Dorogostaisky, Rubtsov & Vlasenko, 1935

**sg. *Wilhelmia*** Enderlein, 1921

*Simulium equinum* (Linnaeus, 1758)

= *zetlandense* (Davies, 1966)

***STEGOPTERNA*** Enderlein, 1930

*Stegopterna trigonium* (Lundström, 1911)

= *richteri* (Enderlein, 1930)

## Excluded species

*Simulium fuscipes* (Fries, 1824) see Notes

*Simulium lineatum* (Meigen, 1804) misidentified, most likely *Simulium equinum*

*Simulium meigeni* Rubzov & Carlsson, 1965 see Notes

= *pygmaeum* of some authors

*Simulium tsheburovae* (Rubtsov, 1956) see Notes

## Notes

***Simulium fuscipes* (Fries, 1824)**. Adler and Crosskey (2014) treat fuscipes as a valid species of *Simulium ruficorne* species group in the subgenus *Nevermannia*, but no known records exist from Finland.

***Simulium meigeni* Rubzov & Carlsson, 1965**. Adler and Crosskey (2014) recognize the identity of *Simulium meigeni* within the subgenus *Hellichiella*, but there are no known records of this species in Finland. The name *pygmaeum* in Kuusela (1971) refers to the form of *pygmaeum* that is a synonym for *pusillum*, not *meigeni* (see Adler and Crosskey 2014).

***Simulium tsheburovae* (Rubtsov, 1956)**. No confirmed records of this species exist from Finland (Raastad et al. 2010), and the name probably subsumes one or more of the existing species within the subgenus *Hellichiella* (Adler and Crosskey 2014).
